# A Digital Template for the Generic Multi-Risk (GenMR) Framework: A Virtual Natural Environment

**DOI:** 10.3390/ijerph192316097

**Published:** 2022-12-01

**Authors:** Arnaud Mignan

**Affiliations:** 1 Institute of Risk Analysis, Prediction and Management (Risks-X), Academy for Advanced Interdisciplinary Studies, Southern University of Science and Technology (SUSTech), Shenzhen 518055, China; mignana@sustech.edu.cn; 2 Department of Earth and Space Sciences, Southern University of Science and Technology (SUSTech), Shenzhen 518055, China

**Keywords:** multi-hazard, accumulation risk, geometric modeling, morphometry, cellular automaton, virtual reality, digital twin, world simulation, complex earth system

## Abstract

Extreme disasters, defined as low-probability–high-consequences events, are often due to cascading effects combined to amplifying environmental factors. While such a risk complexity is commonly addressed by the modeling of site-specific multi-risk scenarios, there exists no harmonized approach that considers the full space of possibilities, based on the general relationships between the environment and the perils that populate it. In this article, I define the concept of a *digital template* for multi-risk R&D and prototyping in the Generic Multi-Risk (GenMR) framework. This digital template consists of a virtual natural environment where different perils may occur. They are geological (earthquakes, landslides, volcanic eruptions), hydrological (river floods, storm surges), meteorological (windstorms, heavy rains), and extraterrestrial (asteroid impacts). Both geological and hydrological perils depend on the characteristics of the natural environment, here defined by two *environmental layers*: topography and soil. *Environmental objects*, which alter the layers, are also defined. They are here geomorphic structures linked to some peril source characteristics. Hazard intensity footprints are then generated for primary, secondary, and tertiary perils. The role of the natural environment on intensity footprints and event cascading is emphasized, one example being the generation of a “quake lake”. Future developments, à la *SimCity*, are finally discussed.

## 1. Introduction

Probabilistic multi-risk assessment, which consists in quantifying multiple risks and their potential interactions, has yet to be fully formalized [1,2,3,4,5]. Multi-risk must additionally become a focus of research and development (R&D) since losses due to cascading effects can be very high relative to the direct losses of an initial event, sometimes exceeding direct losses by several orders or magnitude (e.g., 2005 Hurricane Katrina [6], 2008 Wenchuan earthquake [7], 2010 Eyjafjallajokull volcanic eruption [8], 2011 Tohoku earthquake [9], 2012 Hurricane Sandy [10], 2017 Hurricane Harvey [11], COVID-19 pandemic [12]).

Secondary perils of earthquakes (fires, tsunamis, landslides), of tropical cyclones and other windstorms (surges, inland floods, landslides), and of severe convective storms (hail) are already often considered in catastrophe risk modeling, so is business interruption due to the aforementioned perils [13]. However, tertiary effects, as varied as “quake-lakes” (e.g., Wenchuan earthquake [14]), contingent business interruption (e.g., COVID-19 supply chain interruptions [12]), blackouts (e.g., Hurricane Sandy [15]), or social unrest [16], to only cite a few, have yet to be implemented in a common framework. Many site-specific analyses of various chains-of-events are available [17,18] but methods and results are difficultly transferable to other sites due to the heterogeneity of the models proposed and other silo effects. Multi-risk frameworks have been proposed in recent years [19,20,21,22,23] but share similar issues with methods tailored to a limited set of scenarios constrained by the particularities of each peril [23]. They also remain limited in terms of the number of perils and interactions, relative to the wide space of possible cascading effects [24,25]. Indeed, a comprehensive multi-risk analysis would require modeling—in any given region—the entire chain-of-events crossing the natural, technological, and socio-economic systems.

There is therefore a need for the development of a generic, harmonized, and transparent approach to multi-risk assessment. This is the primary goal of the Generic Multi-Risk (GenMR) framework, a probabilistic multi-risk platform that generates chains-of-events and related risk metrics, based on a Monte Carlo simulation procedure with an adjacency matrix of event interaction at its core [26]. It also naturally includes random co-occurrences of events, but also the memory of previous states which can further amplify risk via dynamic vulnerability for instance [27], as well as non-stationary processes such as seasonality [28]. GenMR was conceived within the context of the European project MATRIX, one of the main international projects on multi-risk of the 2010s [4]. However, due to the intrinsic challenges of the task, GenMR has only been developed in gradual steps and remains incomplete to this day. The work has been pursued by the author episodically over the scope of a decade and remains a long-term initiative. Past developments include the basic GenMR framework with initial tests on abstract data models [26], an application on a generic hydro-dam [28], an application for large earthquake clustering and damage-dependent vulnerability [27], and various other subprojects dedicated to the description of the GenMR adjacency matrix. Those include the exploration of the space of possibilities in cascading disasters within and across the natural, technological, and socio-economic systems (~20 perils) [25], the estimation of conditional probabilities for chains-of-accidents in the oil and gas sector [29], and methods to build and fill the adjacency matrix using expert judgment, reasoned imagination, and wisdom of the crowd [24,30]. Another subproject investigated how GenMR could be integrated under the umbrella of multi-risk governance [3,5,31]. The most recent development was the categorization and harmonization of hazard assessment for individual perils occurring in the natural, technological, and socio-economic systems (~20 perils) [32].

In this article, I describe a *digital template* to be used for R&D and prototyping on GenMR, and which can be considered the last missing piece to generically simulate multi-risk scenarios. This digital template consists of a virtual environment where events may occur and interact. It must be realistic enough to correctly model multi-risk processes while simple enough to convey the richness of plausible multi-risk processes in an intelligible manner. Here, only the natural environment is described; the technological (built) and socio-economic environments will be added at a later stage, which will then allow to implement any type of peril [32] and interaction [25].

**Figure 1:**
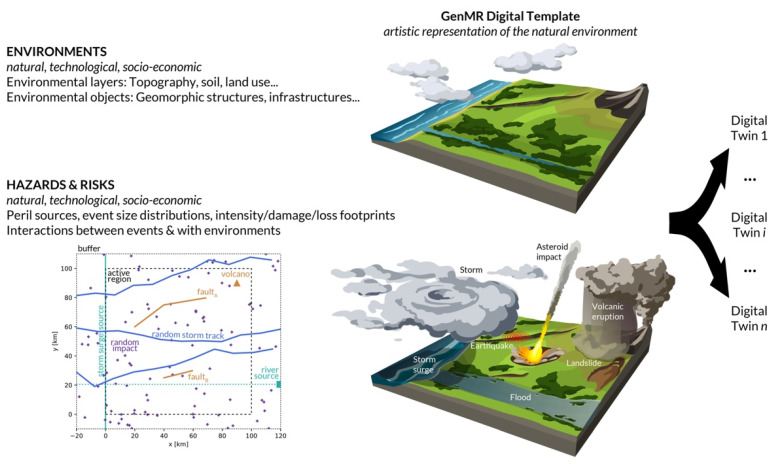
Concept of digital template in the context of the Generic Multi-Risk (GenMR) framework. The version defined in the present article consists of two natural environmental layers (topography and soil) and of some geomorphic structures, as well as of the hazard intensity footprints of the following perils, in alphabetic order: asteroid impacts (AI), earthquakes (EQ), landslides (LS), river floods (RF), storm surges (SS), volcanic eruptions (VE), and windstorms (WS)—For a simplified, earlier version originally coined virtual city, see [3,5,20]. Both previous and new versions are based on an original sketch drawn by the author during the MATRIX project. Peril source locations for the default parameterization (Table A2) are shown on the left map.

Figure 1 illustrates the concept with an artistic representation of the proposed virtual natural environment. The natural perils which occur in this microcosm are of the following nature (see Table A1 for the color scheme): geological (earthquakes, landslides, volcanic eruptions), hydrological (river floods, storm surges), meteorological (windstorms, heavy rains), and extraterrestrial (asteroid impacts). Individual hazards and hazard interactions may depend on the characteristics of the natural environment, especially on the topography and soil conditions. The proposed (i.e., default) parameterization was originally defined by the author within the scope of the MATRIX project with the digital template at the time coined *Virtual City* [3,5,20]. The overarching goal of the GenMR digital template, once completed, will be twofold: (1) to test complex multi-risk scenarios and identify new multi-risk research directions to improve disaster prevention and resilience, and (2) to facilitate future site-specific multi-risk studies by switching between different data models, i.e., moving from a common digital template to different *digital twins* [33,34,35].

The article is structured as follows: The Methods section (Section 2) conceptualizes the digital template, describes how the natural environment can be defined relative to geophysical and hydrological peril sources, and finally lists simple models for hazard intensity assessment. The Results section (Section 3) shows some examples of hazard intensity footprints in the virtual environment for the perils previously mentioned, including secondary and tertiary consequences: storm → surge, heavy rain → (landslides + river flood), earthquake → landslides → river flood (i.e., “quake lake”). The many improvements to be made in the next stages of development of the digital template are then detailed in the Discussion section (Section 4) before some concluding remarks being given in Section 5.

## 2. Methods

This section describes the models and parameterizations needed to generate the virtual natural environment (Section 2.1), as well as the hazard intensity models of the perils that will populate the environment (Section 2.2). Due to the complexity of the processes involved, many simplifications are made in agreement with the generic nature of GenMR. The models and parameters could, however, be changed with no impact on the general purpose of the proposed digital template. The implementation of more sophisticated models and improved parameterizations will be discussed in Section 4. Since this study aims at showcasing the role of a realistic natural environment for the modeling of cascading effects, only multi-hazard scenarios are described in Section 3. The probabilistic component of multi-hazard assessment (i.e., event occurrence rates and conditional probabilities of occurrence) is not included in this study.

### 2.1. Natural Environment Definition

**Figure 2:**
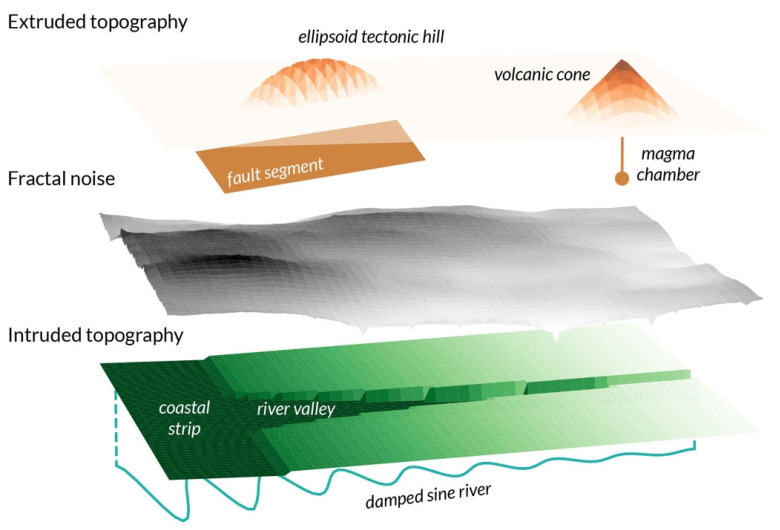
Generation and modification of an environmental layer (here the topography) by intruding and extruding various environmental objects. These objects, which may be associated to peril sources (Table 1), must be included to properly model the chains-of-events that are subject to spatial correlations. An example is the interaction between earthquakes, landslides, and floods under the constraint of local topography.

The proposed digital template could, by definition, take an infinite number of configurations. For illustration purposes, a template is developed that resembles the natural environment shown in Figure 1. The full parameterization is provided in Table A2 and Table A3. The digital template is composed of environmental layers and objects. An *environmental layer* corresponds to any parameter (set) θ defined in a spatial grid (x,y). The primary layer will be the topography with altitude z(x,y). A second one will be soil thickness h(x,y). These layers can be described by additional spatially varying parameters, for example, the slope ϕ(x,y) and geographic aspect for the topography, or some physical characteristics of the soil. *Environmental objects* populate the digital template and can modify environmental layers in function of their characteristics. Peril sources can be related to such objects. Rules of geometric solid modeling [36] and of morphometry [37] will be combined to generate both the layers and objects. The process consists in extruding (i.e., adding) and intruding (i.e., subtracting) solids to other primitive solid “building blocks” [36]. Examples will include a volcanic cone extruding, and a river valley intruding, a background topography. This is illustrated in Figure 2. Development of such a natural environment is necessary for the realistic modeling of mass movement (of soil for landslides, of water for floods), as well as of the spatial correlations leading to event interactions (e.g., a landslide can be triggered by an earthquake due to the mountainous landscape induced by active tectonics).

The peril source type classification, which follows [32], is given in Table 1 alongside the relationships to environmental objects. Fault segments, rivers, the floodable coastline, and volcanos are hence associated to tectonic hills, valleys, the coastal strip, and volcanic edifices, respectively. Hazard intensity footprints will be physically constrained by these objects. Asteroid impact sites and windstorm tracks are independent of the environment defined here, and their locations assumed to be random or pseudo-random. The characteristics of the sources and of the digital template’s spatial grid are listed in Table A2. To simplify the parameterization, the coastline, if defined, has an overall North-South direction, and is located on the Western side of the active region at $x_{0}$ (as in Figure 1). This also constrains the overall slope of the region which must dip westward for water in the entire area to drain toward the coastal boundary.

The map in Figure 1 shows where the various peril sources are located within the default digital template, prior to the definition of any environmental layer or object. Examples using other parameterizations will later be shown to illustrate the many forms that the digital template can take. Note that two regions are defined, an active region which is relevant for multi-risk analysis (dashed box) and a buffer region where sources may also be located, and boundary artifacts might occur (dotted box). By convention, the N-S buffer band of width $\left[x_{min},x_{0}\right]$ represents the mass of water from where storms and storm surges originate. The coordinates of the fault segments, volcano, and river source are given in Table A2. Asteroid impact locations are sampled from a uniform distribution and storm tracks generated as pseudo-random walks (see Table A2 for more details). Developments of more sophisticated versions of the digital template will be discussed in Section 4. The spatial configuration is such that many perils concentrate in a relatively small area prone to interactions.

#### 2.1.1. Environmental Layer 1: Topography

**Figure 3:**
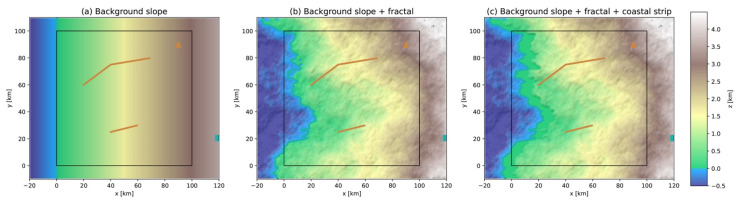
Creation of the background topography for the parameter values given in Table A3. (**a**) Background slope for water evacuation on the western side (step 1); (**b**) fractal topography addition (step 2); (**c**) coastal strip intrusion (step 3). The black box represents the active region. Fixed peril source locations are the same as in Figure 1 and follow the color coding of Table A1.

The first environmental layer to be defined is the topography z(x,y). By convention, its slope must dip westward for water evacuation into the sea or ocean. It is developed in three steps, as shown in Figure 3:

1. *Background slope* (Figure 3a): A plane $z_{bg}\left(x,y\right)$ is defined, tilted westward with slope $\phi_{bg}$ and altitude $z_{bg}\left(x_{0}\right)=0$ (by convention to delimitate the coastline). The slope tangent is here fixed to $\tan\phi_{bg}=3/100$ so that the background altitude increases to 3000 m at the eastern boundary of the active region $x_{1}=100$ km. Such a slope is characteristic of coastal mountains. Using a much smaller slope would be characteristic of a coastal plain.
2. *Fractal topography* (Figure 3b): A fractal topography $z_{f}\left(x,y\right)$ is superposed to the background topography to add some roughness to the otherwise smooth plane, by using the Diamond-square algorithm [38] with fractal dimension $D_{f}=2.6$ [39]. The maximum amplitude of $z_{f}$ is then constrained by the ratio $\eta_{f}$ so that $\max|z_{f}|=\eta_{f}\max z_{bg}$.
3. *Coastal strip* (Figure 3c): A coastal strip of width $w_{cs}$ and slope $\phi_{cs}$ (with $\phi_{cs}<\phi_{bg}$) intrudes the topography $z_{bg}\left(x,y\right)+z_{f}\left(x,y\right)$ at $x_{0}<x\leq x_{0}+w_{cs}$, resulting in a scarp at $x_{0}+w_{cs}$. The width is fixed to $w_{cs}=10$ km and the tangent of the slope to $\tan\phi_{cs}=0.001$, taken from the low range of values given in a global dataset of nearshore slopes [40].

All the parameters are listed in Table A3. They are fixed to be realistic and in range values promoting high hazard intensities, such as mountainous slopes to trigger landslides and very mild nearshore slopes for extended coastal flooding. Notice that the coastline does not remain a straight N-S line once fractality is added, with the abscissa defined at each y as the first x for which $z\left(x\right)\to 0^{+}$. The environmental objects defined in the next subsections are added to, or subtracted from, the background topography between steps 2 and 3.

#### 2.1.2. Environmental Objects Linked to Geological Perils

**Figure 4:**
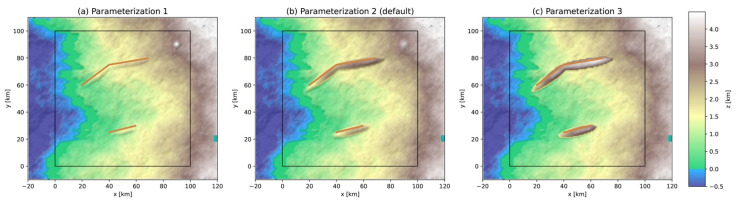
Generation of a volcano and of hills due to active thrust faulting. Tectonic hills are modeled by an ellipsoid extruding the background topography and the volcano is modeled as an extruding cone. (**a**) Parameterization 1: $\Delta z_{hill}=-1.5$, $h_{volc}=2$ km and $w_{volc}=6$ km; (**b**) Parameterization 2 (digital template’s default parameterization): $\Delta z_{hill}=-0.5$, $h_{volc}=1$ km and $w_{volc}=9$ km; (**c**) Parameterization 3: $\Delta z_{hill}=+0.5$ km, $h_{volc}=0.5$ km and $w_{volc}=15$ km. The volcano symbol is hidden to clearly show the modeled volcano. The parameter values were changed to illustrate the process of topography extrusion, and do not necessarily represent realistic estimates of real structures.

Tectonic hills are generated where active thrust fault segments are located. A hill is modeled as an ellipsoid with its axes a function of the fault segment dimensions while the altitude of its centroid (originally centered on the fault segment) can be tuned by the user-defined parameter $\Delta z_{hill}$ to create a hill with realistic characteristics (Figure 2). The axes are fixed to $\left(l_{fault}/\sqrt{2},w_{fault}/\sqrt{2},w_{fault}/(2\sqrt{2})\right)$ where $l_{fault}$ is the fault segment length and $w_{fault}$ the width. As such, only the upper portion of the ellipsoid extrudes the background topography. Examples are shown in Figure 4 for fault segments located at a depth of $z_{fault}=-2$ km. Creation of such an environmental object is particularly important for implementing the correlation between faults, terrain slope, and landslides [41]. For the default value $\Delta z_{hill}=-0.5$ km, the hills are ~1500 m high with a median slope of 21° and maximum slope of 43°, which is consistent with tectonic hill morphometric data [42].

A volcanic edifice is modeled as a cone extruding the background topography with basal width $w_{volc}=9$ km and height $h_{volc}=1$ km, which represent the median estimates from a morphometric study of a global database of composite volcanos [43]. Additional parameterizations are shown in Figure 4 for illustration purposes.

#### 2.1.3. Environmental Objects Linked to Hydrological Perils

**Figure 5:**
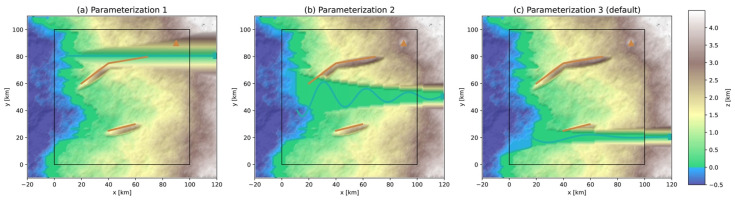
Generation of the river valley composed of a damped sine river and of a flat valley within the exponential contour of Equation (1). (**a**) Parameterization 1: V-shaped valley defined by $A_{river}=0$ km and $\tan\phi_{valley}=1/4$ for a river source located at $y_{RF}=80$; (**b**) Parameterization 2: Flat valley defined by $A_{river}=20$ km, $\varphi_{river}=0.02$, $\omega_{river}=0.2,$ and $\tan\phi_{valley}=1/2$ for $y_{RF}=50$; (**c**) Parameterization 3 (*digital template*’s default parameterization): Flat valley defined by $A_{river}=10$ km, $\varphi_{river}=0.03$, $\omega_{river}=0.1,$ and $\tan\phi_{valley}=1/2$ for $y_{RF}=20$. The parameter values were altered to illustrate the role of Equation (1) on topography intrusion, and do not necessarily represent realistic estimates of real structures.

A river valley is generated, constrained by the coordinates of the river. The general orientation of the structure is West-East and centered on the ordinate of the river flood source $y_{RF}$. The river’s coordinates $\left(x_{i,river},y_{i,river}\right)$ are defined from a damped sine wave with

(1) $$y_{i,river}=A_{river}\cdot \exp\left(-\varphi_{river}\cdot x_{i,river}\right)\cdot \cos\left(\omega_{river}\cdot x_{i,river}\right)+y_{RF},$$

where $A_{river}$ is the amplitude relative to $y_{RF}$ on the coastline (i.e., $x_{0,river}=x_{0}=0$), $\varphi_{river}$ is the decay constant, and $\omega_{river}$ is the angular frequency. The river intrudes the topography with the W-E slope $\phi_{river}<\phi_{bg}$, here fixed to $\phi_{river}=\phi_{cs}$. For any given abscissa x, we have $z=z(x_{river})$ within the ordinate range constrained by the exponential decay contour of Equation (1), forming a flat (alluvial) valley. The intrusion of the topography is not abrupt but is done at a N-S valley-side slope $\phi_{valley}$ (Figure 2). This parameter can take a wide range of values and depends on the cohesion of the soil [44]. Note that $A_{river}=0$ leads to a V-shaped valley. Equation (1) and the associated rules above were chosen to represent the role of the river channel on land surface erosion with the valley changing from a V-shape in the mountainous area to a wider flat-floored valley near the coast with meandering stream, via a transitionary U-shape. Different parameterizations are depicted in Figure 5, with Figure 5c illustrating the final topography of the default digital template, once all the operations described from Section 2.1.1, Section 2.1.2 and Section 2.1.3 are implemented. Notice the resemblance with the original artistic representation shown in Figure 1. It should also be noted that the order of operations on topography extrusion and intrusion match the temporal scale of the different processes involved: Mountain forming first with tilted background topography and fractal topography, followed by recent tectonic hill formation, then erosion by the river, and finally coastal erosion.

#### 2.1.4. Environmental Layer 2: Soil

**Figure 6:**
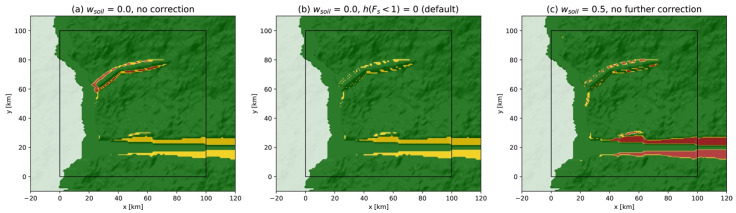
Factor-of-safety maps: (**a**) $h\left(x,y\right)=h_{0}=10$ m and $w_{soil}=0$; (**b**) Default parameterization with correction $h\left(x,y|F_{s}<1\right)=0$ m; (**c**) Extreme case with $w_{soil}=0.5$ and no further change in h(x,y). Stable ($F_{s}>1.5$ ), critical ($1\leq F_{s}\leq 1.5$ ), and unstable ($F_{s}<1$ ) areas are represented in green, yellow, and red, respectively. Large unstable areas are considered unrealistic in the soil layer prior to the generation of any event.

The second environmental layer is the soil thickness h(x,y), initiated to a constant value $h_{0}$. Additional soil characteristics include the effective cohesion $C_{soil}$, effective friction angle $\varphi_{soil}$, density $\rho_{soil}$, and long-term wetness $w_{soil}$ defined as the ratio between the column of water $h_{w}$ and $h_{0}$. Depending on the topography and soil parameterizations, some areas may be unstable and thus prone to collapse. This is evaluated by mapping the factor of safety

(2) $$F_{s}=\frac{\frac{S_{C}}{S_{\rho }gh}+\cos\phi \left(1-S_{w}\frac{\rho_{w}}{S_{\rho }}\right)\tan S_{\varphi }}{\sin\phi },$$

with g is the gravitational acceleration, $\rho_{w}=$ 1000 kg/m^3^ is the unit weight of water, and ϕ is the slope angle [32,45]. With Equation (2) representing the ratio of resisting forces to driving forces, an unstable slope is represented by the condition $F_{S}<1$. Figure 6a shows that unstable areas (in red) are limited to some of the boundaries of the objects introduced to the background topography, principally around tectonic hills. Since these zones are limited in area, the soil thickness is simply fixed to $h\left(x,y|F_{s}\left(x,y\right)<1\right)=0$ m at those grid cells, hence defining some local scarps while removing unstable patches (Figure 6b). To illustrate an unrealistic case, the soil wetness can be increased to $w_{soil}=0.5$, leading to the $F_{S}$ map shown in Figure 6c. It indicates that the soil on the valley flanks is unstable in these new conditions. The landslide cellular automaton model of Section 2.2.3 could then be applied to redistribute the soil, to obtain a more realistic soil distribution, and therefore avoid $F_{S}<1$. Even if such a correction is not necessary for the default parameters (Table A3), the soil distribution could be made more realistic by making h(x,y) a function of the slope and curvature of the topography [46]. This approach is not presented in this article as preliminary tests indicated that it does not significantly impact the triggering of landslides for the default parameterization. Figure 6b shows that areas prone to landslides (in yellow for $1\leq F_{S}\leq 1.5$) are the valley flanks and the hills formed by the active faults.

### 2.2. Hazard Intensity Footprint Modeling

The hazard intensity footprints are generated following the three classes of models described in [32]: Analytical expressions (Section 2.2.1), threshold models (Section 2.2.2), and numeric models (i.e., cellular automata, Section 2.2.3).

#### 2.2.1. Analytical Expressions of Static Event Spatial Diffusion

Static intensity footprints, a simplification of the underlying dynamic process, take the form

(3) $$I\left(x,y\right)=\{\begin{array}{cc} f\left(S,r\right) & \text{for point- and line-sources}\\ \underset{t}{\max}f\left(S,r,t\right) & \text{for track-sources} \end{array},$$

function of the event size S and of the shortest distance to the event source r. This approach here applies to asteroid impacts, earthquakes, volcanic eruptions, and windstorms. The resulting footprints are independent of the environmental layers previously defined. The addition of amplifying factors due to site-specific conditions will be discussed in Section 4.

Asteroid impact footprints are defined by the blast overpressure p [kPa]

(4) $$I_{AI}\left(x,y\right)=p\left(x,y\right)=\frac{1772}{r_{*}^{3}}-\frac{114}{r_{*}^{2}}+\frac{108}{r_{*}},$$

with the dimensional scaled distance $r_{*}=r/\sqrt[{3}]{m_{TNT}}$, the distance to the source r [m], and the event size the explosive mass $S=m_{TNT}$ [kg] [32,47]. Equation (4), originally developed for industrial accidents, is consistent with footprints defined in asteroid impact probabilistic risk models [32,48,49].

Earthquake footprints are defined by the peak ground acceleration (PGA) [cm/s^2^]

(5) $$I_{EQ}\left(x,y\right)=PGA\left(x,y\right)=b_{1}+b_{2}M+b_{3}M^{2}+\left(b_{4}+b_{5}M\right)\log\sqrt{r^{2}+b_{6}^{2}}+b_{7}c_{soft}+b_{8}c_{stiff}+b_{9}c_{N}+b_{10}c_{R},$$

with M is the earthquake magnitude, r [km] is the minimum distance to the fault’s surface trace, $c_{soft}=1$ for soft soil (otherwise 0), $c_{stiff}=1$ for stiff soil (0 otherwise), $c_{N}=1$ for normal faulting, and $c_{R}=1$ for reverse faulting. The empirical parameters are $b_{1}=1.43525$, $b_{2}=0.74866$, $b_{3}=-0.0652$, $b_{4}=-2.7295$, $b_{5}=0.25139$, $b_{6}=7.74959$, $b_{7}=0.0832$, $b_{8}=0.00766$, $b_{9}=-0.05823,$ and $b_{10}=0.07087$ [50]. Based on the proposed natural environment, the soil and tectonic conditions are fixed to $c_{soft}=1$, $c_{stiff}=0$, $c_{N}=0,$ and $c_{R}=1$. The earthquake magnitude is constrained by the rupture length $l_{EQ}$ [km] with $M=4.49+1.49\log_{10}l_{EQ}$ [51]. For a rupture length shorter than the fault segment length, the stochastic rupture initiates at a random point along the fault segment (i.e., floating rupture).

Volcanic eruption footprints are defined by the tephra thickness h [m]

(6) $$I_{VE}\left(x,y\right)=h\left(x,y\right)=h_{VE}\exp\left(-\log(2)\frac{r}{R}\right),$$

where $h_{VE}$ [m] is the maximum thickness and R [m] the half-distance. For a circular footprint

(7) $$R=\log\left(2\right)\sqrt{\frac{V}{2\pi h_{VE}}},$$

where the event size is the volume of tephra S=V [32,52].

Windstorm footprints are defined by the Holland model for tropical cyclones [53] with the windspeed [m/s] defined as

(8) $$I_{WS}\left(x,y\right)=v\left(x,y\right)={\left(\frac{B_{WS}}{\rho_{air}}\left(p_{n}-p_{c}\right){\left(\frac{R}{r}\right)}^{B_{WS}}\exp\left[-{\left(\frac{R}{r}\right)}^{B_{WS}}\right]+\frac{1}{4}r^{2}C^{2}\right)}^{\frac{1}{2}}-\frac{1}{2}rC,$$

where r [km] is the distance to the cyclone center, R [km] is the radius of maximum winds, $p_{c}$ [Pa] is the cyclone’s central pressure, $p_{n}$ [Pa] is the ambient pressure outside the cyclone, $\rho_{air}=$ 1.15 kg/m^3^ is the air density, $1\leq B_{WS}\leq 2.5$ is the Holland $B_{WS}$ parameter, and C [rad/s] is the Coriolis parameter function of the latitude ϕ. The radius of maximum winds is calculated from $\log R=4.0441-1.2090\times 10^{-2}\left(p_{n}-p_{c}\right)+7.2694\times 10^{-3}\phi$ [54]. The latitude is here fixed to ϕ=30° and $B_{WS}=2$ for the default digital template. For a given event size defined by the maximum windspeed $S=v_{max}$, $p_{c}$ is estimated from the equation

(9) $$v_{max}\left(t\right)=\sqrt{\frac{B_{WS}\left[p_{n}-p_{c}\left(t\right)\right]}{\rho_{air}e}},$$

with e Euler’s number [32,53]. $v_{max}$ is assumed to remain constant along the track when the storm is over the water mass ($x<x_{0}\left(y\right)$) and to decrease exponentially once inland [55] with $v_{max}\left(x>x_{0}\right)=v_{max}\left(x_{0}\right)\exp\left(-0.1/v_{WS}\Delta \left(x_{i,track}-x_{0}\right)\right)$, where $v_{WS}$ is the velocity of the storm moving forward. Note that the term windstorm (WS) is used generically.

#### 2.2.2. Threshold Models of Passive Event Emergence

Some hazard intensity footprints can be generated by applying a threshold on an environmental layer. This is the case of the simplest model for storm surge, called the “bathtub” model [32,56]. The event intensity is here defined along the coastline as the water column height $h_{SS}\left(y\right)$ [57], which is the function of the storm windspeed v(y) along the coastline. A simple analytical expression is the polynomial function of the form

(10) $$h_{SS}\left(y\right)=c_{1}v\left(x_{0}\left(y\right)\right)+c_{2}v^{2}\left(x_{0}\left(y\right)\right)+c_{3}v^{3}\left(x_{0}\left(y\right)\right),$$

where $x_{0}$ is the digital template’s coastline abscissa at ordinate y and $c_{1}$, $c_{2}$, and $c_{3}$ are site-specific empirical parameters. The parameter values $c_{1}=0.031641$, $c_{2}=0.00075537$, and $c_{3}=3.1941$ are used for illustration purposes [58]. The surge intensity is then the water height

(11) $$I_{SS}\left(x,y\right)=h_{w}\left(x,y\right)=h_{SS}\left(y\right)-z\left(x,y\right)>0\;\mathrm{or}\;h_{w}\left(x,y\right)=0\;\mathrm{otherwise},$$

which corresponds to the projection of the water surface onto the topography. Projection is done along the *x*-axis only since the present digital template has a simple topography which is oriented W-E.

#### 2.2.3. Numerical Models of Dynamic Event Propagation

For hazard processes which cannot be simplified by an analytical expression or a threshold model, numerical modeling is required. The simplest approach is the cellular automaton, applied here to landslides and river floods, as described in [32]. Both processes correspond to some mass flowing downward on the topography layer z(x,y) under the effect of gravity. The hazard intensity is defined as the maximum height of material (soil or water, respectively) attained at any given location throughout the propagation process, with $I\left(x,y\right)=\underset{t}{\max}I\left(x,y,t\right)$, similarly as in Equation (3).

The propagation of a landslide can be modeled as a Sandpile [59] with the mass to be transferred taken from the soil environmental layer h(x,y). The simplest case consists of initiating mass movement in cells $\left(x_{0},y_{0}\right)$ of unstable slope, which is defined by the condition $F_{S}\left(x,y\right)<1$ (see Equation (2)). The mass is transferred downward to the Moore neighbor of maximum gradient $\left(x_{1},y_{1}\right)$, so that

(12) $$\{\begin{array}{c} \begin{array}{c} z\left(x_{0},y_{0}\right)\to z\left(x_{0},y_{0}\right)-\Delta h\\ h\left(x_{0},y_{0}\right)\to h\left(x_{0},y_{0}\right)-\Delta h \end{array}\\ \begin{array}{c} z\left(x_{1},y_{1}\right)\to z\left(x_{1},y_{1}\right)+\Delta h\\ h\left(x_{1},y_{1}\right)\to h\left(x_{1},y_{1}\right)+\Delta h \end{array} \end{array},$$

where Δh is the column of soil (per geographical cell of width $\Delta_{xy}$) defined as

(13) $$\Delta h=\min\left(\frac{1}{2}\left[z\left(x_{0},y_{0}\right)-z\left(x_{1},y_{1}\right)\right]-w\tan\phi_{stable},h\right),$$

with $\phi_{stable}$ the maximum slope angle ϕ for which $F_{S}\geq 1.5$. Since $h\left(x_{1},y_{1}\right)$ increases, $F_{S}\left(x_{1},y_{1}\right)$ can cross the instability threshold, hence further propagating the landslide. There are two potential triggers, heavy rain and ground shaking, which alter the factor of safety value. Note that once a landslide has occurred, the soil environmental layer h(x,y) is updated according to Equation (12). When h(x,y)=0, a scarp forms with no soil available for further mass movement.

The initiation of a landslide is done by a change in the value of the factor of safety $F_{s}$. Triggering by heavy rain (during a rainstorm) is the simplest case with the column of infiltrated water $h_{w}$ changing the wetness $w_{soil}=h_{w}/h_{0}$ in Equation (2). The intensity of heavy rain could in turn be related to the intensity of a windstorm. Landslide triggering by earthquake ground shaking is modeled by using the Newmark stability method [60]. One possible empirical relationship is

(14) $$\log_{10}D_{N}\left(x,y\right)=-2.71+\log_{10}\left[{\left(1-\frac{a_{c}\left(x,y\right)}{I_{EQ}\left(x,y\right)}\right)}^{2.335}{\left(\frac{a_{c}\left(x,y\right)}{I_{EQ}\left(x,y\right)}\right)}^{-1.478}\right]+0.424M,$$

where $D_{N}$ is the Newmark displacement [m], M is the earthquake magnitude, and $I_{EQ}\left(x,y\right)$ is the matching PGA footprint [m/s^2^] [61]. The parameter $a_{c}$ is the critical acceleration, a function of the terrain characteristics, such that

(15) $$a_{c}\left(x,y\right)=\left[F_{s}\left(x,y\right)-1\right]g\sin\phi ,$$

with $F_{s}$ defined in Equation (2). Mass movement is then initiated at locations where $D_{N}$ crosses a given threshold (here fixed to 1 m). The cellular automaton governed by Equations (12) and (13) is then applied with $\phi_{stable}$ the maximum slope angle ϕ for which $F_{S}\geq 1.5$, estimated by defining a “stable acceleration”

(16) $$a_{stable}\left(x,y\right)=\left[F_{s}\left(x,y\right)-1.5\right]g\sin\phi ,$$

and replacing $a_{c}$ by $a_{stable}$ in Equation (14) [32].

The propagation of a flood is initiated at the location of the river flood source $\left(x_{max},y_{RF}\right)$ with height $h_{w}=Q_{p}\Delta t/\Delta_{xy}^{2}$ where $S=Q_{p}$ is the peak discharge, and Δt is the time interval between two simulation steps. To avoid flooding for $Q_{p}\leq Q_{river}$, the normal river discharge, the altitude at the coordinates of the river $\left(x_{i,river},y_{i,river}\right),$ is reduced by a small amount to keep the mass flow channeled in the river. A simple cellular automaton can be defined with the following rules:

1. Define the absolute height (or motion cost) as the sum of the altitude and water height $h_{tot}=z+h_{w}$;
2. Calculate the gradient (or weight) between the central cell and von Neumann neighbor cells (zero weight for neighbors with equal or greater $h_{tot}$);
3. Discharge the central cell with (some of) the water distributed to the neighbor cells, depending on their weight.

The motion cost can include soil characteristics, such as roughness and infiltration potential. The weights for water distribution are a function of the motion cost at the central and neighbor cells [62], which, in the simplest case, is proportional to the normalized gradients. The simulation stops when all the surplus water has reached the coast. In the current version of the digital template, for convenience, the river flood source takes as inflow the water collected in an implicit catchment basin of area $A_{RF}$ located east of $x_{max}$. It leads to the peak discharge at $\left(x_{max},y_{RF}\right)$ to be a function of heavy-rain intensity $I_{HR}$ defined as a water column. If the precipitation is assumed uniform in both the implicit catchment basin and the digital template, a precipitation component $h_{w}\left(x,y\right)=I_{HR}$ can be added for each cell (x,y) (i.e., pluvial flood) with part of the water draining into the river valley. Pluvial flooding is not tested in the present study. Note that the present model could also be used to simulate the storm surge intensity by defining $Q=h_{w}w\sqrt{gh_{w}}$ where w is the coastline segment’s width [32,57].

Another case of river flooding can be due to environmental changes linked to a landslide altering the topography of the river valley. The topography layer z(x,y) depends on changes in the soil thickness h(x,y) with dz/dt=dh/dt as shown in Equation (12). River flooding is modeled using the cellular automaton just described, in the present scenario considering the constant normal discharge $Q_{river}$ at the most upstream point of the river $\left(x_{max},y_{RF}\right)$. For the unaltered topography, the water level would remain constant along the river and exit on the coastline. If the topography is modified and the flow blocked by an increase of the altitude at a point along the river, the water could not evacuate and would thus accumulate upstream and form a “quake lake”.

## 3. Results

This section showcases examples of hazard intensity footprints in the natural environment of the proposed digital template. Section 3.1 describes the case of the primary perils, which are here independent of the environmental layers. These perils are asteroid impacts, earthquakes, windstorms (here tropical cyclones), and volcanic eruptions. Section 3.2 describes the case of secondary and tertiary perils, which are here dependent of the environmental layers that define the digital template. These perils are storm surges, landslides, and river floods. Regarding river flooding, it could be defined as a primary peril with the peak discharge directly defined for a given point along the river [32] but it is here described as the consequence of heavy rain, alongside rain-triggered landslides.

For each peril, three different event sizes S will be considered for illustration purposes. The following results will prove the importance of a realistic virtual environment for multi-risk R&D.

### 3.1. Intensity Footprints of Primary Perils, Here Independent of the Environmental Layers

**Figure 7:**
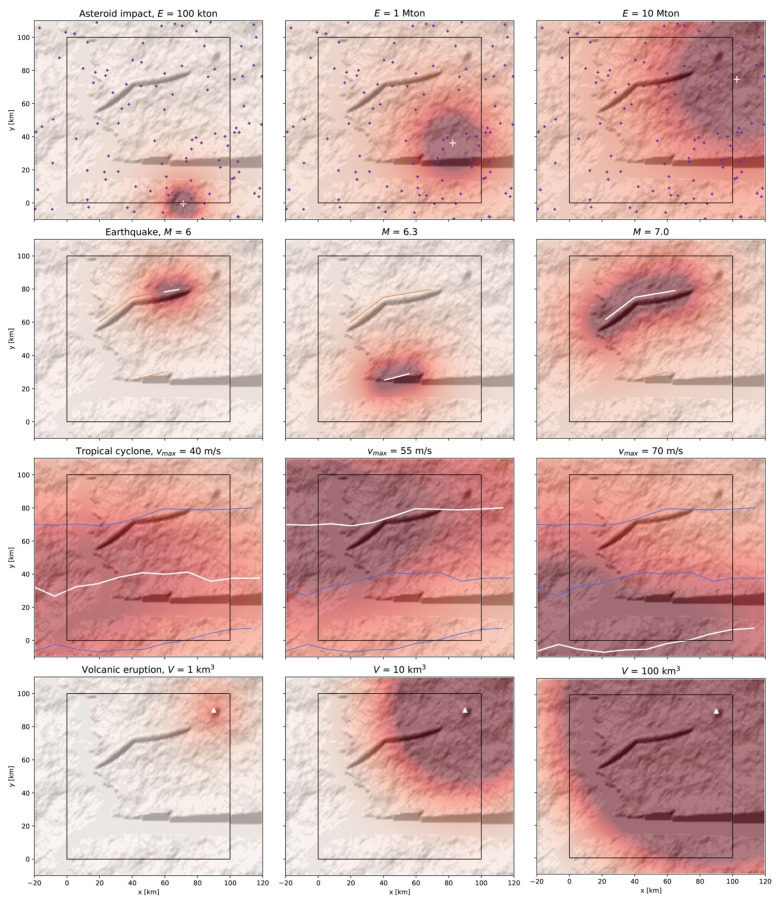
Examples of static intensity footprints for primary perils, based on simple spatial diffusion equations (Section 2.2.1): Asteroid impacts (with kinetic energy E= 100 kton, 1 Mton, or 10 Mton), earthquakes (with magnitude M= 6, 6.3, and 7), tropical cyclones (with maximum windspeed $v_{max}=$ 40, 55, or 70 m/s), and volcanic eruptions (with erupted volume V= 1, 10, or 100 km^3^). In the simplest configuration in which the hazard intensity is not locally modified by site-specific conditions, the footprints are independent of the underlying topography and soil environmental layers. The color scale saturates at intensities for which heavy damage can be expected: at 1 psi of overpressure for asteroid impacts, 0.4 g of PGA for earthquakes, 45 m/s windspeed for tropical cyclones, and 0.5 m of tephra thickness for volcanic eruptions. White segments and white symbols represent the matching event sources. Other sources are colored by peril category, following Table A1.

Intensity footprints based on the models defined in Section 2.2.1 are first generated. Examples of intensity footprints for different event sizes S are shown in Figure 7. For asteroid impacts, 100 random impact sites are shown, and intensities given for three events (AI1, AI2, and AI3), with kinetic energy E= 100 kton, 1 Mton, and 10 Mton, respectively. For comparison, the kinetic energy released by the 2013 Chelyabinsk event was ~500 kton and by the 1908 Tunguska airburst, ~3–50 Mton [63]. The maximum earthquake size in the digital template is constrained by the maximum fault length [51,64]. For smaller event sizes, only part of the fault segment(s) ruptures. The three examples (EQ1, EQ2, and EQ3) shown in Figure 7 have a magnitude M= 6, 6.3, and 7 and occur on fault A, fault B, and fault A, respectively (Figure 1, Table A2). The three windstorms (WS1, WS2, and WS3), here tropical cyclones, have a maximum windspeed $v_{max}=$ 40, 55, and 70 m/s along the track prior to landing. They correspond to events of Category 1, 3, and 5, respectively, on the Saffir–Simpson Hurricane Wind Scale. Finally, the three volcanic eruptions (VE1, VE2, and VE3) occur on the unique volcano of the digital template with erupted volumes V= 1, 10, and 100 km^3^, respectively. Infamous events of comparable sizes are the 1980 VEI-5 Mount St. Helens eruption, the 1991 VEI-6 Pinatubo eruption, and the 1815 VEI-7 Tambora eruption, respectively (with VEI the Volcanic Eruptivity Index).

As previously mentioned, occurrence rates are not defined in the present study. One could use some of the event size distributions given in [32] to rank each event per likelihood. This would, however, require specifying each peril activity in comparison to existing regions of the world. It can already be indicated that asteroid impacts would be the rarest events in the digital template if considering the region to be both seismically and volcanically active. However, while the maximum possible size for most perils is constrained, there is virtually no upper bound for extreme asteroid (and comet) impacts.

All the footprints shown in Figure 7 are simple since they only depend here on the source characteristics. The possible addition of site-specific correcting factors (amplifying or reducing intensity I) as a function of environmental layers will be discussed in Section 4. Storm footprints and earthquake footprints will be used as input to model multi-hazard scenarios in Section 3.2. Asteroid impact and volcanic eruption footprints are only given for completeness in view of a future digital template that will include all potential perils [25,32]. Their relationships to the natural environment and role in cascading effects will, however, be discussed in Section 4. An additional peril not explicitly modeled here is heavy rain. The intensity $I_{HR}$ will be simply assumed to be a constant value of a water column $h_{w}$ for flood and landslide triggering in Section 3.2.

### 3.2. Intensity Footprints of Secondary Perils, Here Dependent of the Environmental Layers

**Figure 8:**
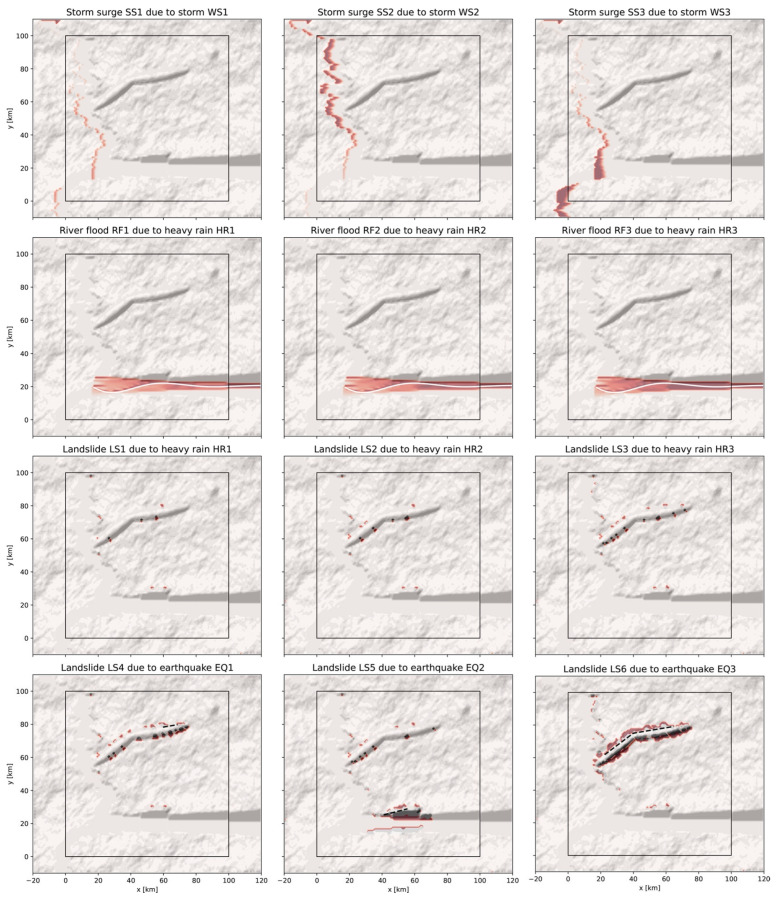
Examples of intensity footprints for secondary perils, based on a simple threshold model for storm surge (Section 2.2.2) and on cellular automata for landslides and river floods (Section 2.2.3). All the footprints here depend on the topography z(x,y) and/or soil h(x,y) environmental layers. The color scale saturates at intensities of 3 m for storm surges, 50 cm for river floods, and 1 m for landslides. The white curves represent the river and the black dashed lines the earthquake segments that trigger the landslides.

**Figure 9:**
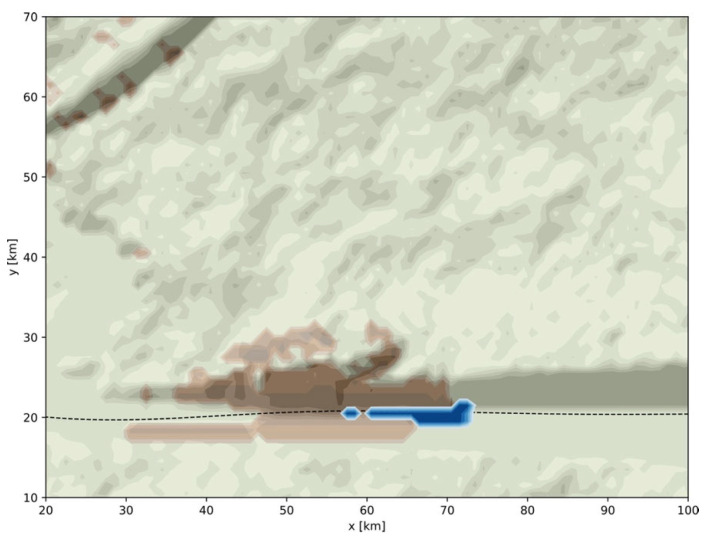
Detail of part of the digital template where a “quake lake” is generated, result of the cascade: earthquake EQ2 → landslides LS2 → river flood FL4. Realized for a modified parameterization of the digital template ($A_{river}=2$ km, all other parameters remaining the same). The displaced soil is represented in brown and the accumulated water in blue.

Intensity footprints based on the models defined in Section 2.2.2 and Section 2.2.3 are now generated. In all cases, the intensity depends on the environmental layers previously defined in Section 2.1. Here, all events are also the consequence of a previous event. These are surges due to storms, river floods, and landslides due to heavy rains, landslides due to earthquakes, and finally “quake lakes”. Three examples of each interaction type are shown in Figure 8, except for the “quake lake” case which will be illustrated in Figure 9.

Storm surges SS1, SS2, and SS3 (first row) are derived from the three storm footprints WS1, WS2, and WS3, respectively, previously shown in Figure 7 (third row) by following Equation (10). Variations in coastal flooding are due to the combined effects of the storm track direction and the topography. The coastal inundation can extend several kilometers inland due to the mild slope $\phi_{cs}$.

River floods RF1, RF2, and RF3 (second row) and landslides LS1, LS2, and LS3 (third row) are due to heavy rain events HR1, HR2, and HR3, respectively. Each rain event is defined by the precipitable water value $h_{HR}=$ 25, 50, or 75 mm/h. Rain is assumed to occur homogeneously in the implicit catchment region east of the digital template with an area here fixed to $A_{RF}=100\times 100$ km^2^ for a duration $\Delta t_{HR}$ of 24 h. The value $h_{w}=h_{HR}\cdot \Delta t_{HR}$ directly modifies the factor of safety $F_{s}$ (Equation (2)) by updating the wetness $w_{soil}\left(x,y\right)=h_{w}/h\left(x,y\right)$, while the flood event size is determined from $Q_{p}\left(x_{max},y_{RF}\right)=h_{w}\cdot A_{RF}/\Delta t_{HR}\;$. Therefore, for the same heavy rainfall, tuning the catchment area can change the flood size independently of the landslide size. For the default parameterization, most of the valley is observed to be inundated, especially the wide floodplain near the coast. Rain-triggered landslides remain very limited in size and occur on the tectonic hills and on the N-S scarp east of the coastal strip.

Landslides LS4, LS5, and LS6 (fourth row) are due to earthquakes EQ1, EQ2, and EQ3, respectively. The soil wetness is fixed to $w_{soil}=0$ (Table A3) and the PGA footprints of Figure 7 (second row) are used as input, following the rules of Equations (14)–(16). Here, landslides occur on the tectonic hills with more local landslides observed than in the heavy rain scenarios. For the case EQ2 → LS5, one can notice some landslides reaching parts of the valley where the river flows (which scenario will be adapted below to generate a “quake lake”).

The impact of the change of the topography z(x,y|LS2) on the normal river flow $Q_{p}\left(x_{max},y_{RF}\right)=Q_{river}$ is finally investigated by re-applying the flood cellular automaton. To interfere with the river flow, the width of the valley is tapered by reducing the amplitude of the damped sine wave (Equation (1)) to $A_{river}=2$ km. All other parameters remain the same as above. Results are shown in Figure 9 with the landslide represented in brown and the flooding, or “quake lake” (RF4), in blue. This example of a chain of three events illustrates the importance of a realistic digital template for the analysis of such a multi-hazard scenario.

## 4. Discussion

I showed that developing a digital template has the potential to improve multi-risk R&D by providing the flexibility of a virtual world that is fully parameterizable. The current version remains very limited in terms of environmental layers and environmental objects defined, as well as of model complexity. Yet, there is so far no equivalent multi-risk strategy being proposed in the scientific literature.

Despite the lack of a simulated built environment and of agent dynamics in this version (see below), the digital template may still be contextualized within a larger world-simulation framework. One early example is the city-simulation *SimCity* videogame [65,66], and the open-source clone of the original 1989 version called *Micropolis* [67,68]. The game includes several perils, such as earthquakes, floods, tornados, fires, nuclear meltdowns, and some exotic peril (monster attacks), alongside smaller-scale events, such as criminality and traffic congestion. Additional perils were added in later versions, including asteroid impacts and (zombie) outbreaks among others. Investigation of the source code of *Micropolis* [67] indicates that event intensity and damage were not based on physics; instead, the occurrence of an event had a binary impact on selected spatial tiles, with all assets remaining intact or being destroyed. Visual inspection of the most recent versions may suggest that the approach remains the same despite a much-improved graphical interface and far more functionalities. The videogame *Cities: Skylines*, similarly to *SimCity*, simulates disasters but it remains unclear if their intensity and impact on the built environment are in part based on physics. This videogame was built with the Unity Game Engine [69], which is also used to develop digital twins [70]. Some “serious disaster videogames” exist [71], including *Stop Disasters!* [72] and others based on *Cities: Skylines* [73], but they focus on disaster risk reduction education and/or crisis management, not on the physics of catastrophes and of the underlying environment. Proposals to use *SimCity*-like games remain so far limited to emergency planning practice [74], and in recent years, to the development of reinforcement learning strategies [68]. It seems therefore worthwhile to further develop a virtual world that simulates both events and the environment that they populate based on physical considerations. In view of the overwhelming task at hand, which is to implement at the very least two dozen perils and their interactions within and across the natural, technological, and socio-economic systems [25,32], many simplifications will have to systematically be made in accordance with the generic nature of GenMR.

Planned improvements to the natural environment of the GenMR digital template include the definition of a land-use environmental layer to distinguish vegetation from built areas and agricultural land. It will have a direct impact on pluvial flooding (via different water infiltration levels) and landslides (with a modified $F_{S}$ model to include the role of tree root systems) for instance. Additional perils will be added, such as wildfires and later, urban fires [32]. Ecological disasters could also be added in the future, such as crop failure [32]. The parameters of the soil environmental layer will also be able to vary in space and interact with volcanic eruptions, with the physical characteristics of volcanic ash potentially facilitating mass movements on the volcanic edifice (i.e., lahars). Different conditions on soil and surface roughness could also modify ground shaking [75], windspeed [76], and water flow [77], locally. To address the impact of climate on hazards and their interactions, an atmospheric environmental layer should be added, with temperature, pressure, and water vapor as some of the main variables. Seasonality has already been implemented in GenMR [28] but has yet to be systematically modeled in year-simulations.

The development of the technological environment will follow, with the characterization of the built areas and agricultural lands as economic assets (exposure layer) and the definition of new environmental objects corresponding to critical infrastructures. Associated perils, of which simple hazard models are already described in [32], will include industrial explosions, blackouts, and hydro-dam failure [28]. The spatial distribution of assets will be based on simple rules of land planning by using cellular automata once again [78,79] and allometry [80]. Again, spatial correlations between environmental layers and event interactions can be expected (e.g., urban growth attracted to water and mild topography [78,79]). The final layers to model will be associated to the socio-economic environment, where businesses and groups of people will be defined as environmental objects in the exposure layer. Here, many lessons shall be learned from *SimCity*-like videogames which are themselves designed as cellular automata populated with agents [74]. Additional perils, already described in [32], will include business interruption, social unrest, and epidemics. An important aspect will be the filling of conditional probabilities of occurrence in the GenMR adjacency matrix of interactions [25].

In parallel to building the multiple layers of the digital template, the development of more sophisticated models relating peril sources to environmental characteristics will be investigated by using machine learning. Those surrogate models could later replace the simple rules so far proposed in Section 2.1.

The final outputs of GenMR are year-simulations of potential chains-of-events, hazard curves and hazard maps, and various risk metrics, such as the average annual loss (AAL), occurrence exceedance probability (OEP), and aggregate exceedance probability (AEP) curves [26]. A user can test different parameterizations to investigate the impact of cascading effects and their spatiotemporal correlations on risk for improved decision-making. In the context of multi-risk governance [5,31], GenMR corresponds to the multi-risk knowledge generation phase. Transition from a digital template for R&D to digital twins for specific sites or regions will require some integration with the other phases of risk governance, which are the social and institutional context and stakeholder processes [31]. In a first phase, the digital template could already help decision-makers better comprehend the complexity of multi-risk.

## 5. Conclusions

GenMR is a simplified, generic, and—most importantly—fully transparent physics-based framework for multi-risk assessment. Considering the six-layer digital twin model proposed by [70], the virtual environment associated to GenMR will only include the first four layers: terrain, buildings, infrastructures, and mobility. As a digital template, it should help integrating multi-risk considerations into future digital twin cities [34,35,70].

Many improvements will be required (see list in Section 4) to provide a comprehensive multi-risk simulation framework for implementation in digital twins. Until then, the current version of the digital template (and upcoming versions including the technological and socio-economic environmental layers) will already provide a sandbox for multi-risk R&D and further prototyping in the context of the complex Earth system paradigm [81]. The proposed approach, still at its initial stage, already proves its potential, with, for example, the case of a “quake lake” simulation, which exemplified a virtual chain-of-three-events and its interactions with the natural environment (Section 3).

The authors of one of the most recent multi-hazard frameworks [23] mentioned the critical issues of hazard typology heterogeneities and resource limitations to implement many types of input data and models in a same framework. The GenMR digital template, with simulated data (this study) and harmonized models [25,32], should help solve this well-known and long-lasting problem in multi-risk research [1].

## Figures and Tables

**Table 1:** Peril source and related environmental object.

Peril ID	Peril Name	Source	Source Type *	Related Object
AI	Asteroid impact	Impact site	Point	N/A
EQ	Earthquake	Fault segment	Line	Tectonic hill
LS	Landslide	Topography	Diffuse	N/A
RF	River flood	Upriver point	Point	River valley
SS	Storm surge	Coastline	Line	Coastal strip
VE	Volcanic eruption	Volcano	Point	Volcanic edifice
WS	Windstorm	Depression	Track	N/A

* Following the classification proposed by [32].

## Data Availability

Not applicable.

## Appendix A

Table A1 provides the color scheme used to describe different perils by origin, following [32].

**Table A1:** Color scheme per peril category.

Color	Category	Perils
	Extraterrestrial	Asteroid impact
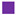
	Geophysical	Earthquake, landslide, volcanic eruption
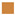
	Hydrological	River flood, storm surge
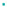
	Meteorological	Tropical cyclone
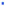

All the parameters used to define the default digital template are listed in the following tables.

**Table A2:** Default parameterization for the spatial grid and peril source characteristics.

Parameter	Value	Description
$\left(x_{0},x_{1},y_{0},y_{1}\right)$	(0,100,0,100)	Active box boundaries [km]
$\left(x_{min},x_{max},y_{min},y_{max}\right)$	(−20,120,−10,110)	Buffer box boundaries [km]
$\Delta_{xy}$	1	Grid cell size [km]
$ID_{fault}$	$\left({\mathrm{fault}}_{A},{\mathrm{fault}}_{B}\right)$	Fault segment label (reverse only)
$w_{fault}$	(5,5)	Fault segment width [km]
$\phi_{fault}$	(45,45)	Fault segment dip [°]
$x_{i,fault}$	((20,40,70),(40,60))	Abscissas of fault segment tips [km]
$y_{i,fault}$	((60,75,80),(25,30))	Ordinates of fault segment tips [km]
$z_{fault}$	(2,2)	Fault segment depth [km]
$\left(x_{RF},y_{RF}\right)$	$\left(x_{max},20\right)$	River flood source coordinates [km]
$A_{RF}$	100×100	Upstream catchment basin area [km^2^]
$\left(x_{volc},y_{volc}\right)$	(90,90)	Volcano coordinates [km]
$x_{0,track}$	$x_{min}$	Abscissa of storm track start (ordinate random uniform in range $\left[y_{min},y_{max}\right]$)
$n_{track}$	10	Number of points along storm track
$l_{track}$	1	Standard deviation of track segment length (total length normalized to $x_{max}-x_{min}$)
$\phi_{track}$	π12	Standard deviation of angle between W-E line and track segment
$B_{WS}$	2	Holland parameter (tropical cyclone)
$v_{WS}$	17	Forward velocity of storm [km/h]

**Table A3:** Default parameterization for the topography and soil layers.

Parameter	Value	Description
$z_{0}$	0	Altitude of background topography at $x_{0}$ [km]
$\tan\phi_{bg}$	3100	Slope tangent of the background topography [1]
$\tan\phi_{cs}$	11000	Slope tangent of the coastal strip [1]
$w_{cs}$	10	Width of the coastal strip [km]
$D_{f}$	2.6	Fractal dimension of the topography [1]
$\eta_{f}$	0.5	Ratio of maximum fractal altitude to maximum background altitude [1]
$\Delta z_{hill}$	−0.5	User-defined vertical shift of the hill ellipsoid centroid [km]
$w_{volc}$	9	Width of the volcanic edifice’s cone [km]
$h_{volc}$	1	Height of the volcanic edifice’s cone [km]
$A_{river}$	10	River damped sine wave’s initial amplitude [km]
$\varphi_{river}$	0.03	River damped sine wave’s decay constant [1]
$\omega_{river}$	0.1	River damped sine wave’s angular frequency [1]
$\tan\phi_{river}$	11000	Slope tangent of the W-E river [1]
$\tan\phi_{valley}$	12	Slope tangent of the N-S river valley intrusion [1]
$Q_{river}$	200	River normal discharge [m^3^/s]
$h_{0}$	10	Initial soil depth [m]
$w_{soil}$	0	Soil background wetness [1]
$C_{soil}$	$20\times 10^{3}$	Soil effective cohesion [Pa]
$\varphi_{soil}$	27	Soil effective friction angle [°]
$\rho_{soil}$	2650	Soil density [kg/m^3^]
